# Effects of Multi-Sensory Stimulation on Brain Functional Connectivity in Patients with Disorders of Consciousness

**DOI:** 10.3390/brainsci16030299

**Published:** 2026-03-07

**Authors:** Jiaxue Tong, Fangfang Sun, Tao Min, Zixuan Chen, Yong Yang

**Affiliations:** College of Automation, Hangzhou Dianzi University, Hangzhou 310018, China

**Keywords:** DOC, CGC, brain functional connectivity, multi-sensory stimulation

## Abstract

**Highlights:**

**What are the main findings?**

- By combining high-density EEG with conditional Granger causality, this work suggests that visual–olfactory combined stimulation may enhance directed functional connectivity in patients with disorders of consciousness (DOC).
- Visual–olfactory stimulation not only activates connections in brain regions corresponding to single-modality stimulation, but may also enhance the exchange of information between brain regions that are not directly stimulated and other brain regions.

**What are the implications of the main findings?**

- These findings reveal how cross-modal collaboration upon stimulation temporarily opens dormant information pathways in patients with DOC, linking sensory neuroscience to clinical recovery.
- The portable, low-cost paradigm is suitable for ICU and rehabilitation settings, offering a bedside approach that can be used alongside or while awaiting invasive interventions.

**Abstract:**

**Background/Objectives:** This study investigates the effects of multi-sensory stimulation on brain functional connectivity in patients with disorders of consciousness (DOC). DOC patients experience prolonged loss of consciousness due to brain injury, posing significant challenges for rehabilitation. **Methods:** In the study, visual, olfactory, and visual–olfactory (V-O) combined stimulation were applied to DOC patients while their EEG signals were recorded. A brain functional network was constructed based on the conditional Granger causality (CGC) method to analyze its topological characteristics. **Results:** The results revealed that the strength of brain functional connectivity in Minimally Conscious State (MCS) patients was significantly higher than that in Vegetative State (VS) patients, indicating a strong correlation between the intensity of synergistic activity in brain functional connectivity and the level of consciousness. Furthermore, V-O combined stimulation significantly enhanced brain functional connectivity compared to single-modality stimulation. The selection of different stimulation also differentially affected brain functional connectivity, with olfactory stimulation exhibiting high pleasure, arousal, and dominance (Self-Assessment Manikin) values demonstrating unique advantages in reducing individual variability and improving global efficiency. **Conclusions:** The study provides a theoretical foundation for the application of multi-sensory stimulation in the rehabilitation of DOC patients. V-O stimulation not only enhances information transmission in brain regions corresponding to visual and olfactory processing under single-modality stimulation but also increases the intensity of information transfer to other brain regions; this may serve as a reference for understanding the effects of multi-sensory stimulation on brain networks.

## 1. Introduction

Disorders of consciousness (DOC) refer to a pathological state in which loss of consciousness persists for more than 28 days due to various types of brain injuries, such as traumatic brain injury, stroke, and hypoxic–ischemic encephalopathy [1,2,3]. It can be specifically classified into Vegetative State (VS), Unresponsive Wakefulness Syndrome (UWS), and Minimally Conscious State (MCS). DOC patients typically suffer from severe neurological damage, accompanied by complex functional impairments and complications. In recent years, significant advancements in neuroimaging, neuroelectrophysiology and neuromodulation techniques have greatly enhanced the understanding and diagnostic methods for DOC. However, due to challenges such as diagnostic complexity, prognostic uncertainty, and limitations in treatment options, the therapeutic efficacy for DOC still lacks clear evidence-based medical support, necessitating prolonged rehabilitation therapy.

Electroencephalogram (EEG) technology can intuitively reveal cognitive functions that DOC patients are unable to behaviorally demonstrate, thereby providing clinicians with reasonable prognostic assessments, early interventions, and clinical decision-making support [4]. Several studies have currently utilized EEG technology to analyze brain network connectivity in DOC patients, scientifically assessing the degree of consciousness impairment and regional brain damage through characteristics of EEG signals from different brain regions. Zhuang et al. [5] explored the relationship between consciousness states and brain network control patterns, finding increased control costs in the brain networks of DOC patients. Modolo et al. [6] investigated methods using extremely low-frequency (ELF) brain stimulation techniques to quantify consciousness levels and proposed an EEG-based potential indicator for assessing consciousness levels. Sun et al. [7] introduced a novel EEG measurement method for detecting states of consciousness, achieving classification of consciousness states in DOC patients. Naro et al. [8] studied functional connectivity under resting-state EEG and discovered that multiplex and multilayer network analysis metrics could better distinguish between different types of DOC patients. Liuzzi et al. [9] used an elastic net regression model to predict consciousness recovery in DOC patients, finding that models combining EEG and clinical data performed best in predicting recovery from disorders of consciousness.

Patients with DOC commonly experience cognitive impairments, and providing them with cognitive stimulation can effectively enhance individual cognitive, social, and communication functions [10]. Wang et al. [11], through fMRI-based brain network analysis, identified the olfactory cortex as a region with significant differences in patients with DOC. As a core hub of the limbic system, the olfactory cortex has dense fiber connections with emotional and memory centers such as the amygdala and hippocampus. This suggests that olfactory stimulation may exert a positive impact on consciousness recovery by activating these structures and promoting the release of neurotransmitters. Hilz et al. [12] clearly demonstrates that olfactory stimulation can serve as a powerful tool for probing and revealing the integrity of complex brain networks involved in emotional and autonomic regulation. Ren et al. [13] provided a brain network perspective, demonstrating that visual stimulation serves as a sensitive probe for assessing the functional state of brain networks and can also modulate multi-scale brain network plasticity through interventions such as light therapy. However, the limitations of unimodal stimulation become apparent when addressing complex symptoms and multifaceted dysfunctions. In contrast, multimodal intervention approaches, which combine various therapeutic measures, can more comprehensively improve patients’ cognitive functions and enhance rehabilitation outcomes [14]. Studies have shown that early, multimodal, and individualized rehabilitation therapy can significantly increase the likelihood of consciousness recovery, improve function and quality of life, promote neuroplasticity, reduce complications, and enhance survival rates [15]. Pape et al. [16] found that multi-sensory stimulation could increase arousal levels in DOC patients and, to some extent, improve their cognitive functions. Chen Shuran et al. [17] summarized advances in the clinical application of multi-sensory stimulation, noting its positive effects on consciousness, perception, cognition, behavior, and emotion in patients with various brain injuries.

Currently, research on brain networks in patients with disorders of consciousness (DOC) under multimodal stimulation is limited. Recently, Min et al. (2025) employed EEG microstate analysis to investigate neural dynamics in patients with DOC during visual, olfactory, and visual–olfactory combined (V-O) stimulation [18]. Their study focused on the spatiotemporal dynamics of microstates, revealing that the gradient attenuation of resting-state microstate D parameters reflects the severity of DOC, and that task-specific responses can distinguish MCS+ patients. Building upon the same database, the present study combines electroencephalography (EEG) with conditional Granger causality analysis to construct brain functional networks in the same cohort of DOC patients under an identical multi-sensory (visual, olfactory, and V-O) stimulation paradigm. The aim is to reveal differences in network topological properties between patients in Minimally Conscious State (MCS) and Vegetative State (VS) under multimodal stimulation, as well as the regulatory effects of different odor attributes on brain network efficiency. These findings are expected to provide a new neurophysiological basis for optimizing multi-sensory rehabilitation protocols for patients with DOC.

## 2. Experiment

### 2.1. Construction of Brain Functional Networks

A brain functional network refers to the interconnected network formed by neural activity interactions between different regions of the brain. These networks are responsible for processing and transmitting information, regulating cognitive, emotional, behavioral, and other complex functions. In neuroscience research, the analysis of brain functional networks is a critical method that helps us understand the working principles of the brain and the mechanisms underlying brain disorders. Studies on brain functional networks are often based on complex network theory, which posits that nodes (i.e., brain regions) and connections (i.e., neural pathways) collectively form networks with specific topological properties [19]. This paper employs the multivariate Granger causality method to calculate causal relationships between multi-channel EEG signals to assess brain functional connectivity in DOC patients.

If the historical information of variable $X_{i,t}$ can significantly improve the prediction of variable $X_{j,t}$, then $X_{i,t}$ is said to have a Granger causal influence on $X_{j,t}$. When the number of time series exceeds two, the influence of other time series must be considered. This study employs the Conditional Granger Causality (CGC) algorithm to analyze causal relationships between any time series under the influence of other variables’ historical information, based on a Vector Autoregression (VAR) model. The VAR model is a statistical model used to capture linear interdependencies among multiple time series variables. Suppose we have k time series:

$$X_{t}\;=\;{\left(X_{1,t},\;X_{2,t},\;\ldots ,\;X_{k,t}\right)}^{T}$$

The VAR model can be expressed as:

$$X_{t}\;={\;A}_{1}X_{t-1}\;+\;A_{2}X_{t-2}\;+\ldots +\;A_{p}X_{t-p}\;+\;\varepsilon_{t}$$

p represents the maximum lag order considered in the model. We performed AIC and BIC analyses on the EEG data of each individual subject to determine their respective optimal lag orders. To ensure that the model adequately captures the dynamic characteristics of all subjects, the maximum value among the individual optimal lag orders was ultimately selected as the global model order, i.e., p = 8. $A_{\tau }$ denotes the k × k coefficient matrices, and $\varepsilon_{t}$ represents the regression estimation residuals. To test whether $X_{i,t}$ has a Granger causal influence on $X_{j,t}$, it is necessary to design a restricted model under the assumption that $X_{i,t}$ has no influence on $X_{j,t}$. In this restricted model, $A_{\tau }^{*}$ represents the constrained form of coefficient matrix $A_{\tau }$, where all coefficients associated with $X_{i,t}$ are set to zero. The procedure is as follows:

$$\left\{\begin{array}{c} X_{t}=\sum_{\tau =1}^{p}A_{\tau }X_{t-\tau }+\varepsilon_{t}\\ X_{t}=\sum_{\tau =1}^{p}A_{\tau }^{*}X_{t-\tau }+\eta_{t} \end{array}\right.$$

$\varepsilon_{t}$ and $\eta_{t}$ represent the regression estimation residuals of the full model and the restricted model, respectively. Their means are zero, and they are mutually uncorrelated, with variances $\sigma_{\varepsilon_{t}}^{2}$ and $\sigma_{\eta_{t}}^{2}$. The causal influence of $X_{i,t}$ on $X_{j,t}$ can be expressed as:

$${\mathrm{CGC}}_{i\to j}\;=\;\ln\frac{\sigma_{\varepsilon_{j}}^{2}}{\sigma_{\eta_{j}}^{2}}$$

**Figure 1:**
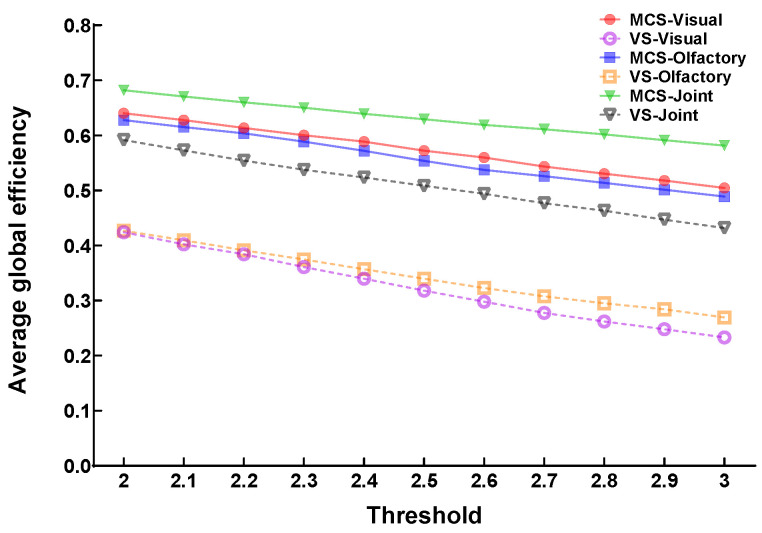
Sensitivity analysis across the threshold range of 2–3 with a step size of 0.1.

Subsequently, an F-test combined with false discovery rate (FDR) correction was applied to obtain a statistically significant Granger causality matrix (*p* < 0.05). The locations of the electrode channels were used as nodes to ensure that there were no isolated nodes in the networks across all participants under different stimulation conditions. At this point, the maximum possible value (threshold = 2.6) was set as the threshold. When the absolute value of an element in the Granger causality matrix exceeded the threshold, a connection edge was established between the corresponding nodes, thereby constructing the corresponding brain functional network. By performing stability analysis on the global efficiency of different patients under various stimulation conditions within the threshold range of 2 to 3 with a step size of 0.1, the results showed that the performance within this threshold range was consistent with the subsequent conclusions, as shown in Figure 1.

### 2.2. SAM Emotional Assessment

**Figure 2:**
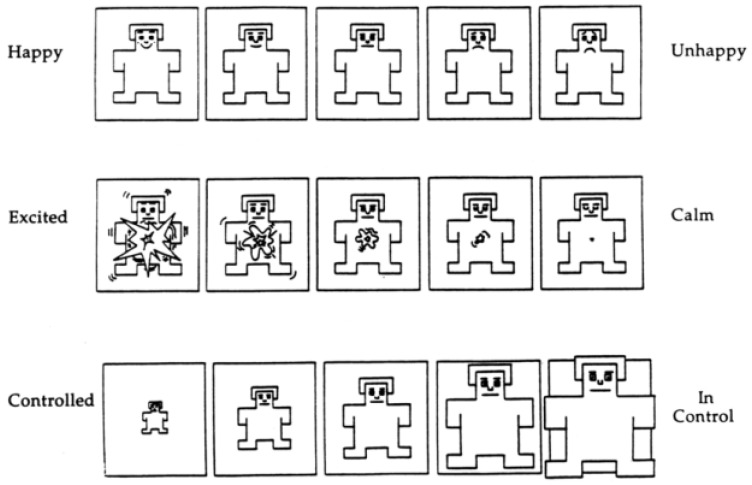
Self-assessment Manikin. From top to bottom, they represent pleasure (P), arousal (A), and dominance (D) respectively.

The experiment invited 17 healthy volunteers to evaluate 24 odor types for satisfaction. The Self-Assessment Manikin (SAM) is an odor emotion scale comprising three dimensions: pleasure, arousal, and dominance. This scale (1—“not at all”, 9—“extremely”) was used to classify emotional levels, as shown in Figure 2. The selected odors (all above the olfactory threshold of healthy populations) were delivered via an odor dispenser developed by ScentRealm (Hangzhou, China) to avoid interference from unstable odor delivery. Each odor had three similar distractors to assess familiarity, with adequate rest periods between odor presentations.

The SAM self-assessment model is a picture-oriented evaluation tool with three dimensions: pleasure (P), arousal (A), and dominance (D) [20]. The pleasure dimension describes the positive or negative nature of an emotion, i.e., whether it is pleasant or unpleasant. High-pleasure emotions are generally considered positive, such as joy and satisfaction, while low-pleasure emotions are typically negative, such as sadness and anger. The arousal dimension describes the intensity or energy level of an emotion, i.e., whether it is arousing or calming. High-arousal emotions may include excitement and fear, while low-arousal emotions may include relaxation and boredom. The dominance dimension describes an individual’s sense of control or power in emotional expression. High-dominance emotions may include confidence and arrogance, while low-dominance emotions may include submission and helplessness.

Given that DOC patients commonly experience cognitive impairments, this study added a familiarity (F) dimension. This dimension describes the degree to which an individual is familiar with a specific object, information, or context during cognitive processing. When encoding familiar information, the brain can recognize and categorize it more quickly and retrieve it more easily when needed [21]. The brain can rapidly identify familiar information, thereby reducing cognitive load and improving processing speed [22]. Familiar information can also influence an individual’s attention allocation [23].

**Figure 3:**
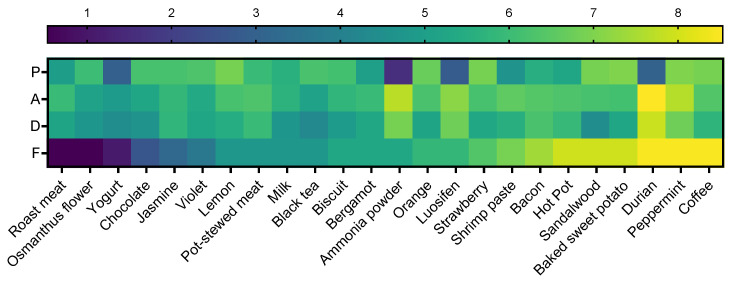
Viridis heatmap of pleasure, arousal, dominance and familiarity for all odors.

To facilitate easier recognition and focused attention on experimental stimulation among patients with disorders of consciousness, this study adopted familiarity as the primary criterion for stimulus selection. The P, A, D values of all odors were visualized using a Viridis heatmap (Figure 3) and ranked based on F. Ultimately, three odors with high F, A, and D values but varying *p* values (both high and low) were selected: hot pot, durian, and peppermint.

### 2.3. Formal Experiment

#### 2.3.1. DOC Participants

The study collected EEG signals from 23 DOC patients. After excluding 5 patients with significant EEG signal interference, standardized causal modeling was performed on the 32-channel EEG signals of the remaining 18 DOC patients (MCS = 8, VS = 10) under different stimulation. The patients’ ages ranged from 34 to 72 years. Among them, there were 10 males (4 with MCS, 6 with VS) and 8 females (4 with MCS, 4 with VS). All subjects were from Beijing Tiantan Hospital. Prior to the experiment, the CRS-R scale was used to assess the level of consciousness in the patients. None had a history of psychiatric disorders or genetic predisposition to psychiatric illnesses, and no medications that could potentially affect normal cognitive judgment were administered before the experiment. This study was performed in line with the principles of the Declaration of Helsinki. Approval was granted by the Ethics Committee of the Beijing Tiantan Hospital, Capital Medical University (KY2023-175-03).

#### 2.3.2. Stimulation Paradigm

**Figure 4:**
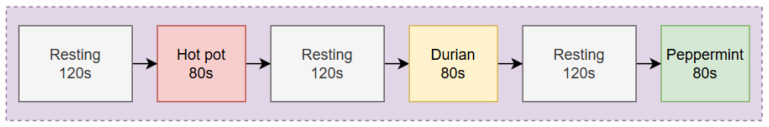
Stimulation paradigm.

Each DOC patient was in good mental condition during the stimulation sessions and sequentially received three types of stimulation: visual, olfactory, and V-O. Each stimulation session lasted 10 min, with a 10 min rest interval between consecutive sessions to rule out potential carryover effects from the previous stimulation. The stimulation paradigm is illustrated in Figure 4.

The EEG data acquisition was performed using PN-NET multi-channel EEG topographer from Beijing Yunshen Technology Co., Ltd. (Beijing, China). Electrodes were arranged according to the 10-10 international system, recording signals from 32 electrodes as listed in Table 1. The reference electrodes were placed on bilateral mastoids, with electrode–scalp impedance maintained below 5 kΩ. The sampling frequency was set at 256 Hz. EEG data were collected from DOC patients in a quiet room when they were in stable condition.

The acquired data underwent preprocessing, during which the electrode signals were re-referenced using the average reference of the whole brain. For non-physiological artifacts, power frequency interference was eliminated through digital filtering, and a band-pass filter of 0.5–50 Hz was applied. For physiological artifacts, independent component analysis (ICA) was employed to remove interference signals such as electrooculographic (EOG), electrocardiographic (ECG), and electromyographic (EMG) artifacts [24].

#### 2.3.3. Statistical Analysis

In this study, F-tests were performed on the computed conditional Granger causality matrices, followed by false discovery rate (FDR) correction to obtain statistically significant Granger causality matrices.

In the statistical analysis of brain network topological parameters (Section 3.3 and Section 3.4), independent-sample *t*-tests were used to compare differences between patient groups (MCS vs. VS) under the same stimulation condition. One-way repeated measures analysis of variance (ANOVA) was employed to evaluate differences within the same patient group across different stimulation conditions, followed by paired *t*-tests for post hoc pairwise comparisons. All *p*-values from these comparisons were corrected using the FDR method.

Prior to analysis, all topological metrics were confirmed to follow a normal distribution using the Shapiro–Wilk test (*p* > 0.05), satisfying the requirements for parametric testing. For the repeated measures ANOVA, Mauchly’s test of sphericity was performed; if the sphericity assumption was violated, the Greenhouse–Geisser correction was applied.

## 3. Results

### 3.1. Construction Results of Brain Functional Networks

**Figure 5:**
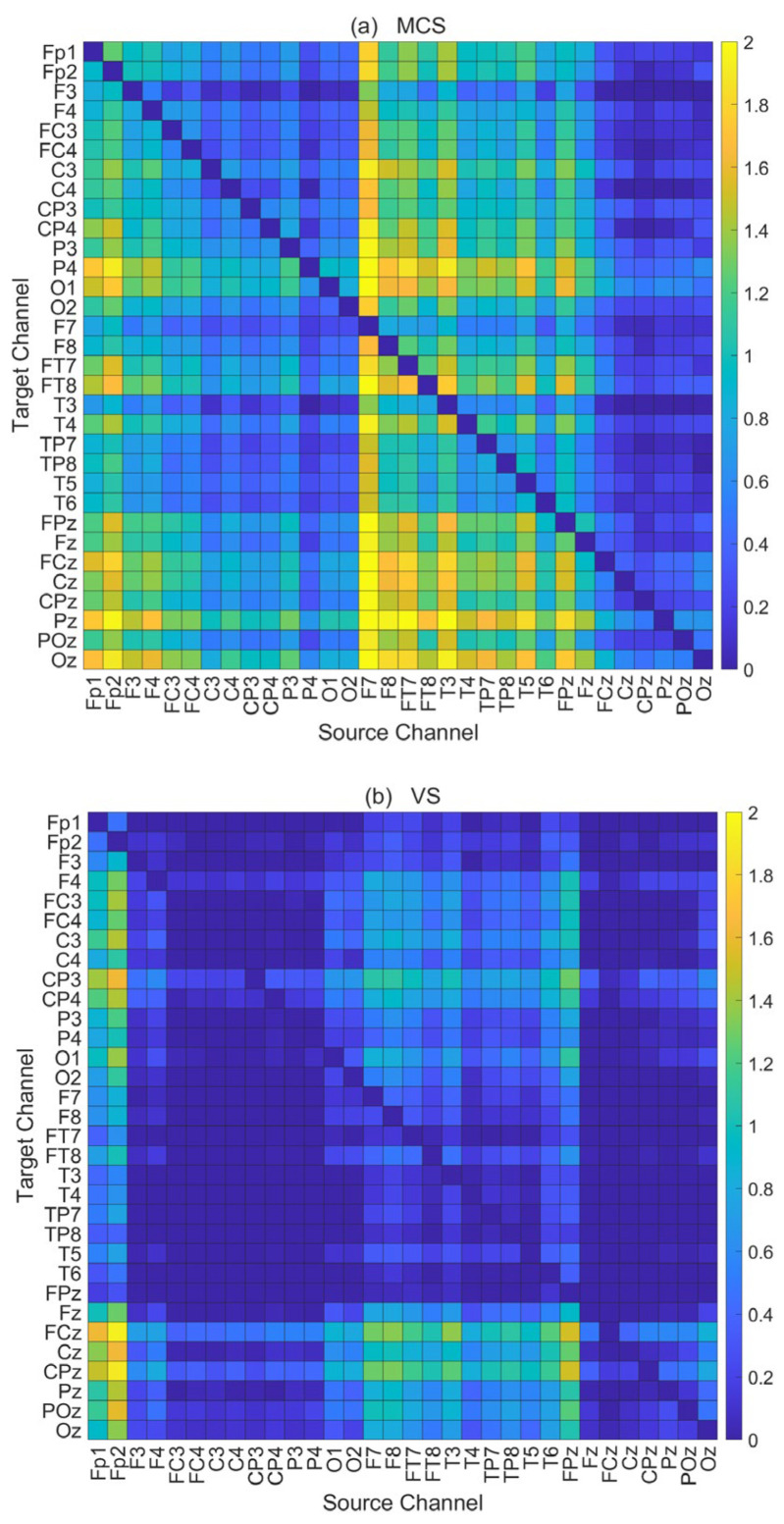
Average multivariate granger causality matrix across all tasks for all subjects under visual-olfactory combined stimulation in MCS and VS patients. (**a**) MCS patients; (**b**) VS patients.

Based on the CGC analysis framework, this study constructed multivariate Granger causality matrices for all DOC patients by calculating directional information flow between channels under each task condition. The average multivariate Granger causality matrices for all tasks under V-O stimulation for both MCS and VS patients are shown in Figure 5. In the matrix, the horizontal axis represents the Source Channel, and the vertical axis represents the Target Channel. The intensity of the Colorbar on the right reflects the causal influence strength from the Source Channel to the Target Channel. Lighter colors and larger values in the matrix indicate stronger causal effects from the Source Channel to the Target Channel. The results show that the overall causal connection density in MCS patients is significantly higher than that in the VS group.

**Figure 6:**
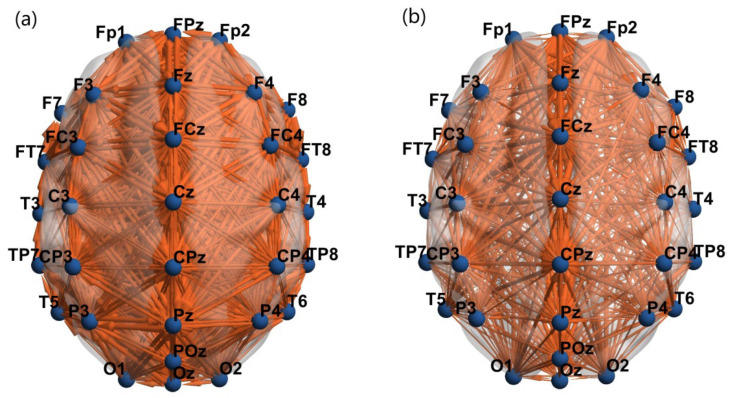
Brain functional connectivity networks in MCS and VS patients under V-O stimulation. (**a**) MCS patients; (**b**) VS patients.

The location of each channel on the brain map was defined as a node. Directed connections were established between corresponding nodes based on the causal relationships between Source Channels and Target Channels in Figure 6, thereby constructing brain functional connectivity networks for MCS and VS patients under V-O stimulation. It was found that the strength of functional connectivity between brain regions in MCS patients was higher than that in VS patients, with the number of directed connected edges in the brain networks of MCS and VS patients being 817 and 473, respectively, representing an increase of 72.73%. The increases in the number of edges for different patient groups under visual and olfactory stimulation were 487.29% and 558.42%, respectively, as shown in Table 2.

### 3.2. Topological Properties of Patients Under Different Sensory Stimulations

Topological features of brain functional networks are important tools for describing and quantifying the structural characteristics of complex networks. The existing studies generally describe whole-brain networks using functional segregation metrics, functional integration metrics, and nodal metrics [25]. These metrics reflect the network’s organization, information transmission efficiency, and node importance from different perspectives.

Functional integration metrics primarily focus on global connections and information transmission efficiency between different regions in the brain network, reflecting the brain’s ability to rapidly integrate information from distributed regions. Common metrics include the average clustering coefficient and global efficiency.

Nodal metrics are mainly used to evaluate the importance and role of individual nodes in the network, reflecting their connectivity and influence. Common nodal metrics include in-degree and out-degree.

In a directed network, the out-degree of a node refers to the number of connections originating from that node and pointing to other nodes, while the in-degree refers to the number of connections coming from other nodes and pointing to that node.

$$\left\{\begin{array}{c} k_{i}^{\mathrm{out}}=\sum_{j=1}^{N}{\mathrm{CGC}}_{i\to j}\\ k_{i}^{\mathrm{in}}=\sum_{j=1}^{N}{\mathrm{CGC}}_{j\to i} \end{array}\right.$$

**Figure 7:**
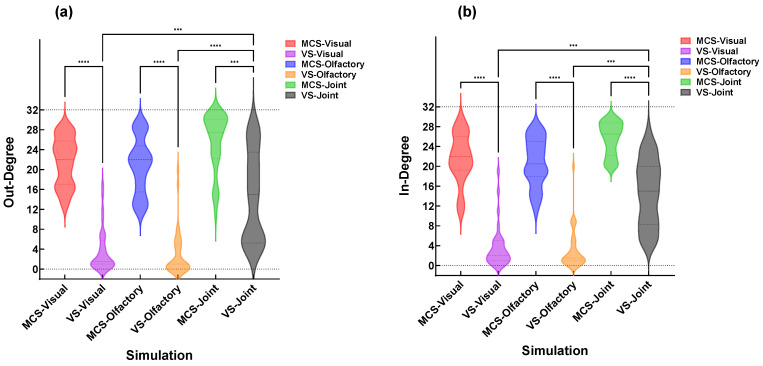
Violin plots of out-degree and in-degree for all DOC patients under different stimulation conditions. (**a**) Out-degree; (**b**) in-degree. (*** *p* < 0.001, **** *p* < 0.0001).

To investigate the information transmission capabilities of different DOC patients under various sensory stimulations, the in-degree and out-degree of all DOC patients under different stimulations were plotted as violin plots (Figure 7). MCS patients performed significantly better than VS patients under all three stimulation conditions. Moreover, during V-O stimulation, both MCS and VS patients exhibited higher in-degree and out-degree values compared to single-modality stimulation, indicating stronger brain information transmission in both groups under V-O stimulation (* *p* < 0.05, ** *p* < 0.01, *** *p* < 0.001, **** *p* < 0.0001).

**Figure 8:**
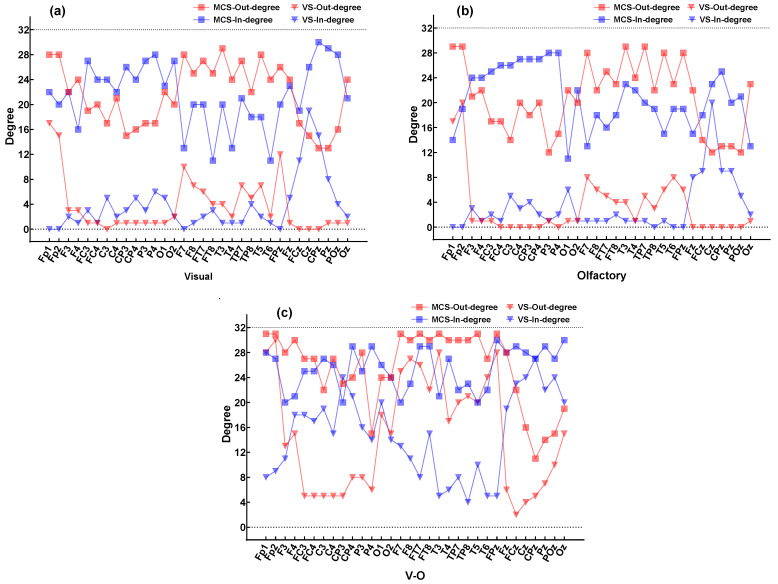
Changes in average of in-degree and average of out-degree across all channels in DOC patients. (**a**) Visual stimulation. (**b**) Olfactory stimulation. (**c**) V-O stimulation.

To analyze differences in the causal flow direction of EEG signals among different DOC patients under various sensory stimulations, the changes in the average in-degree and out-degree across all channels for all subjects are shown in Figure 8. The comparison of the total number of connected edges in the network between MCS and VS patients under different stimulations is shown in Table 2. Under visual and olfactory stimulation, the in-degree and out-degree of each channel in MCS patients were significantly higher than those in VS patients. Notably, under V-O stimulation, both MCS and VS patients showed a significant increase in the in-degree and out-degree across channels, indicating that V-O stimulation engaged more brain regions in information transmission compared to visual or olfactory stimulation alone; patients exhibited stronger brain functional connectivity.

Multi-sensory stimulation directly affects the brain regions receiving the stimulation and influences other regions which are not directly stimulated through cross-modal integration and enhanced neural network connectivity. This effect helps to improve brain’s efficiency and function [26]. Under V-O stimulation, all patients exhibited a substantial increase in the in-degree and out-degree of O1, O2, Oz, Fp1, Fp2, T3, and T4. Among these, O1, O2, and Oz are associated with visual processing, while Fp1, Fp2, T3, and T4 are related to olfactory processing. Additionally, the in-degree of the FC, C, and CP brain regions significantly increased compared to single-modality stimulation. These findings suggest that V-O stimulation may enhance information transmission not only in the brain regions corresponding to visual and olfactory processing, but also to other brain regions, thereby facilitating more effective information transfer within the brain.

### 3.3. Topological Properties of Patients Under Different Stimuli

**Figure 9:**
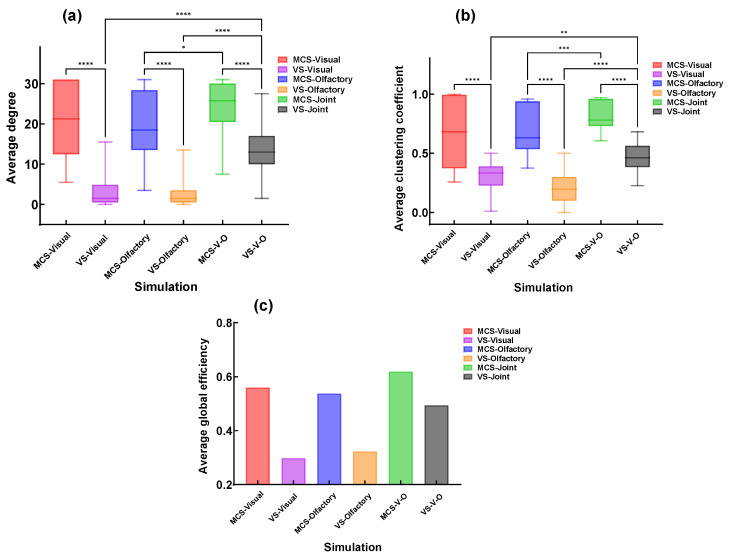
Topological properties of brain functional networks in different DOC patients. (**a**) Average degree. (**b**) Average clustering coefficient. (**c**) Average global efficiency. (* *p* < 0.1, ** *p* < 0.01, *** *p* < 0.001, **** *p* < 0.0001).

To analyze the effects of different sensory stimuli on patients’ brain functional connectivity, the study analyzed and compared three topological parameters—average degree, average clustering coefficient, and global efficiency—of brain functional networks in different DOC patients under three sensory stimulation conditions (Figure 9).


$$D=\frac{1}{N}\sum_{i=1}^{N}\sum_{j=1}^{N}w_{\mathrm{ij}}$$

$w_{\mathrm{ij}}$ represents the connection weight between node i and node j.

The average clustering coefficient is the mean value of the clustering coefficients of all nodes in the network.

$$C=\frac{1}{N}\sum_{i=j}^{N}C_{i}$$

Global efficiency (E_glob_) measures the global efficiency of information transmission within a network and can be used to characterize the global connectivity features of the network.

$$E_{\mathrm{glob}}=\frac{1}{N\left(N-1\right)}\sum_{i,j\in N,i\neq j}\frac{1}{d_{\mathrm{ij}}}$$

$d_{\mathrm{ij}}$ represents the shortest path length between node i and node j, i.e., the minimum number of edges traversed from node i to node j.

The study found that compared to VS patients, MCS patients exhibited higher average degree, average clustering coefficient, and global efficiency. Additionally, visual performance was slightly higher than olfactory. Furthermore, for both MCS and VS patients, these three metrics under the V-O condition were significantly higher than those under visual or olfactory stimulation alone.

### 3.4. Topological Properties of Patients Under V-O Stimulation

**Figure 10:**
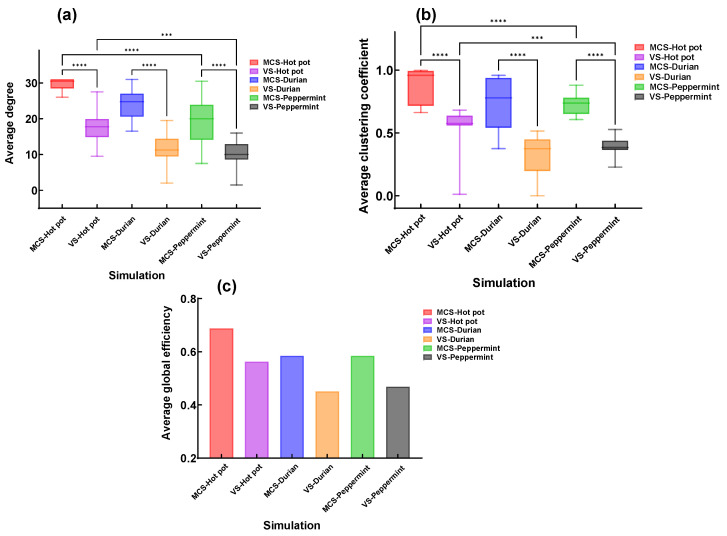
Topological characteristic parameters under V-O stimulation across different DOC patients with various stimulation. (**a**) Average degree. (**b**) Average clustering coefficient. (**c**) Average global efficiency. (*** *p* < 0.001, **** *p* < 0.0001).

To further compare the differential effects of stimulus selection on the improvement of brain functional connectivity in patients with disorders of consciousness (DOC), three topological features—average degree, average clustering coefficient, and global efficiency—were analyzed under different olfactory stimuli combined with the V-O condition (Figure 10). The results showed that MCS patients exhibited higher average degree, average clustering coefficient, and global efficiency than VS patients. The effectiveness of the three stimuli ranked as follows: hot pot, durian, and mint. However, mint stimulation showed a more concentrated distribution in the average clustering coefficient, with smaller individual differences compared to durian stimulation. Additionally, the global efficiency under hot pot stimulation was significantly superior to that of the other two stimuli.

## 4. Discussion

Studies indicate that a widespread and complex time-delay structure exists in the brains of healthy individuals, which is considered the foundation of functional connectivity in brain networks and may be closely related to the emergence of consciousness [27]. In DOC patients, this time-delay structure in the brain alters correspondingly with changes in consciousness levels. Further comparative analysis reveals that the strength of direct connections between different brain regions is closely associated with consciousness levels. For instance, MCS patients exhibit stronger neural connectivity and metabolic activity in the prefrontal and parietal networks compared to VS patients [28,29], suggesting that enhanced synergistic activity between brain regions correlates with higher consciousness levels.

The rehabilitation environment for DOC patients is often monotonous, lacking diverse sensory stimulation and social interactions, which may lead to psychological stress, anxiety, depression, and other emotional issues. These negative emotions not only adversely affect mental health but may also hinder the recovery of cognitive functions and delay rehabilitation progress. Multi-sensory stimulation, compared to single-modality stimulation, can partially simulate the normal living environment of healthy individuals, reducing these negative emotions in patients [30]. It also demonstrates significant advantages in promoting arousal, improving neural function, and shortening coma duration [31]. Based on the CGC algorithm, this study constructed and analyzed causal networks from EEG signals of DOC patients under visual, olfactory, and V-O stimulation. Analysis of brain network topological features revealed that MCS patients exhibited stronger brain functional connectivity than VS patients across all stimulation types, indicating that the intensity of synergistic activity in brain functional connectivity is associated with consciousness levels in DOC patients. For all DOC patients, V-O stimulation significantly enhanced brain functional connectivity compared to single-modality stimulation. Relevant brain regions simultaneously exhibited neural representations for both stimulations, with activation intensities significantly higher than those under unimodal stimulation. More importantly, this combined stimulation mode also promoted information transmission to brain regions not directly stimulated. This finding suggests that cross-modal integration may enhance the processing efficiency of local brain regions, support the formation of complex perceptions by facilitating broader interregional information exchange, and play a greater role in neuroplasticity.

In combined stimulation, the selection of different stimulation produced varying effects on brain functional connectivity. The study found that hot pot scent stimulation resulted in the highest strength of brain functional connectivity, followed by durian scent, and finally peppermint scent. This may be because hot pot is more widely known and is a positive stimulus that can effectively stimulate appetite. However, peppermint scent stimulation demonstrated more concentrated average clustering coefficients, with smaller individual differences. This indicates that while hot pot and durian scents may more strongly activate overall brain functional connectivity, peppermint scent (with high P, A, D values) may be more suitable for individualized stimulation protocols, as it can more uniformly activate patients’ brain networks and reduce inter-individual variability. Future research could further explore the characteristics and mechanisms of different stimulation to optimize multi-sensory stimulation protocols.

## 5. Conclusions

This study integrates multi-sensory stimulation with the functional connectivity of brain networks in DOC patients. Using CGC, we constructed and analyzed differences in brain functional connectivity between patients in MCS and those in VS under visual stimulation, olfactory stimulation, and combined V-O stimulation. Furthermore, we compared and analyzed the effects of different stimulations under V-O stimulation on brain functional connectivity in MCS and VS patients. The results demonstrate that under visual, olfactory, and V-O stimulation, MCS patients exhibited stronger brain functional connectivity than VS patients, indicating an association between brain functional connectivity and the level of consciousness in DOC patients. Compared to unimodal stimulation, both MCS and VS patients exhibited enhanced brain functional connectivity under V-O stimulation, suggesting that multi-sensory stimulation is more likely than unimodal stimulation to promote information transmission in patients with DOC. V-O stimulation may not only elevate the activation levels of the stimulated brain regions but also facilitate information transfer to non-stimulated brain areas, revealing a dual role of cross-modal integration in local enhancement and global brain network coordination.

Although this study yielded meaningful findings, it has certain limitations. First, these findings are suggestive; however, the small sample size of patients with DOC and the lack of a healthy control group limit the generalizability of the results. Furthermore, these findings warrant validation in studies employing a counterbalanced design. Future research should expand the sample size to confirm the conclusions drawn here and further explore differences among other subgroups of patients with DOC. Future studies should expand the sample size to validate the conclusions drawn here and further explore differences among other subgroups of DOC patients. Second, this study only included three types of visual and olfactory stimulation. Future research could incorporate a wider variety and diversity of stimulation, as well as combine other types of sensory stimulation. Additionally, this study only conducted short-term experimental observations on DOC patients. Long-term follow-up studies could be performed in the future to evaluate the prolonged effects of multi-sensory stimulation on consciousness recovery in DOC patients.

## Figures and Tables

**Table 1:** Name of electrode corresponding to each brain region.

Brain Area	Pole Name
Frontal lobe	Fp1, Fp2, F3, F4, FC3, FC4, F7, F8, FpZ, Fz, FCz
Temporal lobe	FT7, FT8, T3, T4, TP7, TP8, T5, T6
Parietal lobe	CP3, CP4, P3, P4, CPz, Pz
Occipital lobe	O1, O2, POz, Oz
Central	C3, C4, Cz

**Table 2:** Comparison of the total number of connected edges in the network between MCS and VS patients under different stimulations. (In a directed network, the total out-degree equals the total in-degree, so the values in the table represent the total number of connected edges in the network).

	Visual		Olfactory		V-O		V-O vs. Visual	V-O vs. Olfactory
MCS	693	487.29%	665	558.42%	817	72.73%	17.89%	22.86%
VS	118		101		473		300.85%	368.32%

## Data Availability

The data presented in this study are available on request from the corresponding author. The data supporting the findings of this study are not publicly available due to the need to protect the privacy of human subjects, which is in line with the ethical approval requirements (Ethics Committee of Beijing Tiantan Hospital Affiliated to Capital Medical University, protocol code: KY2023-175-03).
